# Microbubble formulation influences inflammatory response to focused ultrasound exposure in the brain

**DOI:** 10.1038/s41598-020-78657-9

**Published:** 2020-12-09

**Authors:** Dallan McMahon, Anne Lassus, Emmanuel Gaud, Victor Jeannot, Kullervo Hynynen

**Affiliations:** 1 Physical Science Platform, Sunnybrook Research Institute, Toronto, Canada; 2 Department of Medical Biophysics, University of Toronto, Toronto, Canada; 3 Bracco Suisse S.A., Plan-les-Ouates, Switzerland; 4 Institute of Biomaterials and Biomedical Engineering, University of Toronto, Toronto, Canada

**Keywords:** Permeation and transport, Drug delivery, Blood-brain barrier

## Abstract

Focused ultrasound and microbubble (FUS + MB)-mediated blood–brain barrier (BBB) permeability enhancement can facilitate targeted brain-drug delivery. While controlling the magnitude of BBB permeability enhancement is necessary to limit tissue damage, little work has attempted to decouple these concepts. This work investigated the relationship between BBB permeability enhancement and the relative transcription of inflammatory mediators 4 h following sonication. Three microbubble formulations, Definity, BG8774, and MSB4, were compared, with the dose of each formulation normalized to gas volume. While changes in the transcription of key proinflammatory mediators, such as *Il1b, Ccl2, and Tnf,* were correlated to the magnitude of BBB permeability enhancement, these correlations were not independent of microbubble formulation; microbubble size distribution may play an important role, as linear regression analyses of BBB permeability magnitude versus differential gene expression for these proinflammatory mediators revealed significantly greater slopes for MSB4, a monodisperse microbubble with mean diameter of 4 μm, compared to Definity or BG8774, both polydisperse microbubbles with mean diameters below 2 μm. Additionally, the function of an acoustic feedback control algorithm, based on the detection threshold of ultraharmonic emissions, was assessed. While this control strategy was effective in limiting both wideband emissions and red blood cell extravasation, microbubble formulation was found to influence the magnitude of BBB leakage and correlations to acoustic emissions. This work demonstrates that while the initial magnitude of FUS + MB-mediated BBB permeability enhancement has a clear influence on the subsequent inflammatory responses, microbubble characteristics influence these relationships and must also be considered.

## Introduction

The well-established utility of encapsulated microbubbles as contrast agents in ultrasound imaging has for decades prompted research into their potential therapeutic applications. One such use has been for the targeted delivery of drugs across the BBB. Leveraging their high degree of echogenicity, the combination of intravenous microbubble administration and noninvasive transcranial FUS can be employed to transiently increase BBB permeability in targeted locations^1^, providing an avenue for therapeutic agents to enter the brain from systemic circulation (reviewed in^2,3^). Thus far, phase one clinical trials have employed Definity^4–6^ and SonoVue (used with implantable ultrasound devices)^7,8^ microbubbles, while preclinical work has explored the use of both commercially available contrast agents, like Optison^1^, SonoVue^9^, and Definity^10^, as well as in-house developed formulations^11,12^; however, detailed interrogation of the effects microbubble characteristics have on biological responses, beyond quantifying BBB permeability enhancement and overt tissue damage, is lacking.

The composition and size distribution of microbubbles can vary considerably between formulations, which in turn can affect their response to insonation^13–15^. Typically composed of lipids, proteins, or polymers, the shells of microbubbles act to stabilize a gas core and govern many aspects of their behaviour, including circulation half-life^15^ and probability of collapse^16^. Similarly, their size, generally between 1 and 10 μm in diameter, both allow transit through capillary beds and influence a host of factors (e.g. resonance frequency^17,18^). The distribution of microbubble sizes, polydisperse for most clinically approved formulations, also merits consideration, as greater dispersion will contribute to a wider range of behaviours within a microbubble population at pressures relevant to BBB permeability applications; this motivates the study of microbubble formulations with monodisperse size distributions. The response of microbubbles to insonation in turn influences the types and magnitude of stress exerted on vascular walls^19^. Thus, in the context of FUS + MB-mediated BBB permeability enhancement, the interplay between microbubble formulation and biological responses warrants further study.

Previous work has demonstrated that microbubble size^11,20,21^ and total gas volume^22^ influence the initial magnitude of BBB permeability enhancement generated by FUS + MB exposures; the latter has been proposed as a unifying dose parameter^22^. While establishing factors that contribute to the initial magnitude of BBB permeability enhancement has relevance for predicting drug delivery^23,24^ and treatment safety^25,26^, there has yet to be thorough investigation into whether or not different regimes of BBB permeability enhancement converge to produce similar biological responses. For example, previous work has reported positive correlations between gadolinium contrast enhancement, an indicator of BBB permeability magnitude, and the relative expression of proinflammatory cytokines 6 h following sonication^26^; however, it is unclear whether generating an equivalent increase in BBB permeability enhancement using differing FUS + MB exposure parameters will produce the same inflammatory response. Differences may arise due to the generation of several routes of BBB leakage^27^, all of which contribute on a macroscopic level to quantitative increases in BBB permeability (i.e. increased in gadolinium contrast enhancement), but are driven by distinct biological processes.

The work detailed here explores biological responses following FUS + MB exposures, comparing outcome measures between three microbubble formulations. Relationships between the initial magnitude of BBB permeability enhancement and the transcription of proinflammatory mediators are explored, along with the efficacy of a clinically relevant acoustic feedback control strategy. Ultimately, these results emphasize the necessity to tailor acoustic control strategies to specific microbubble formulations and suggest that factors beyond initial BBB permeability enhancement influence subsequent inflammatory responses.

## Materials and methods

### Animals

Male Sprague Dawley rats (n = 45), weighing 200–350 g on the day of sonication, were used in this study (Taconic Biosciences, Germantown, NY, USA). Animals were housed in the *Sunnybrook Research Institute* animal facility (Toronto, ON, Canada) with access to food and water ad libitum. Three cohorts of animals were used to compare microbubble formulations on measures related to FUS + MB-mediated BBB permeability enhancement. Cohort #1: Microbubble half-life in circulation (n = 9); Cohort #2: Acoustic feedback control algorithm testing (n = 24); Cohort #3: Relationship between BBB permeability enhancement and acute gene expression (n = 12). All animal procedures were approved by the *Animal Care Committee* at *Sunnybrook Research Institute* and are in accordance with guidelines from the *Canadian Council on Animal Care*.

### Microbubbles

For all animal cohorts, three microbubble formulations were assessed: (1) Definity, (2) BG8774, and (3) MSB4. Commercially available Definity microbubbles (Lantheus Medical Imaging, North Billerica, MA, USA) are composed of octafluoropropane gas encapsulated in an outer phospholipid shell and display a polydisperse size distribution. A research grade polydisperse agent, BG8774 (Bracco Suisse S.A., Plan-les- Ouates, Switzerland), and one research grade monodisperse agent, MSB4 (Bracco Suisse S.A., Plan-les-Ouates, Switzerland), were also employed. An overview of the microbubble gas, shell composition, mean microbubble diameter (Dn), polydispersity expressed as the geometric standard deviation (GSD), shell stiffness (χ), and shell viscosity (κ_s_) of the different formulations are presented in Table 1. The viscoelastic shell parameters of Definity were taken from literature^28^, whereas those of the research grade formulations were characterized using acoustic attenuation measurements as outlined by Segers et al.^29^.

**Table 1:** Physicochemical and viscoelastic shell properties of the microbubble formulations.

Microbubble	Shell composition	Gas	*Dn* (µm)	GSD	*χ* (N m^−1^)	*κ*_*s*_ (× 10^−9^ kg s^−1^)
Definity	DPPC/DPPA/DPPE-PEG5k	C_3_F_8_	1.19	1.51	0.8^28^	2.98
BG8774	DSPC/DPPE-PEG5k	C_4_F_10_/N_2_	1.29	1.59	0.41	2.0
MSB4	DSPC/DPPE-PEG5k	C_4_F_10_	4.17	1.07	0.66	5.83

*Dn* mean microbubble diameter, *GSD* geometric standard deviation, *χ* shell stiffness, *κ*_*s*_ shell viscosity.

**Figure 1:**
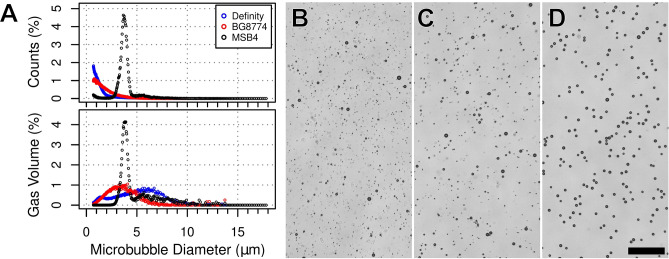
Microbubble size distribution. (**A**) Relative distribution of microbubble count and gas volume are displayed for each formulation. Definity and BG8774 exhibited polydisperse size distributions, each with more than 80% of microbubbles measuring less than 2 μm in diameter. MSB4 displayed a monodisperse size distribution, with approximately 85% of microbubbles measuring between 3 and 5 μm in diameter. Brightfield microscope images, collected with 20 × objective, show the size distributions for Definity (**B**), BG8774 (**C**), and MSB4 (**D**). Scale bar = 50 µm.

Microbubble size distribution, from which Dn, GSD, gas volume, and microbubble concentration were extracted, was assessed using a Coulter Counter Multisizer 3 (Beckman Coulter Inc., Brea, CA, USA) with a 30 μm aperture, allowing a measurable size range of 0.7–18 μm. Isoton II (Coulter Electronics, Luton, UK), passed through a 0.2 μm filter, was used to dilute microbubble samples (1:1000). Measurements were performed at room temperature within 15 min of Definity activation or BG8774 resuspension, or once MSB4 (frozen) had reached room temperature. Background noise measurements were performed prior to each microbubble measurement and found to be negligible. Measurements were performed in triplicate for each microbubble formulation. Coulter counter measurements of each microbubble formulation are displayed in Fig. 1.

Both Definity and BG8774 displayed polydisperse size distributions with approximately 93% and 82% of bubbles measuring less than 2 μm in diameter, respectively. Approximately 85% of MSB4 microbubbles measured between 3 and 5 μm in diameter. Definity, BG8774, and MSB4 all contained less than 1% of microbubbles larger than 7 μm in diameter. For all in vivo experiments and microbubble formulations, a gas volume equivalent to 20 μL (liquid volume)/kg of Definity, was administered intravenously.

### Animal preparation

For all animal cohorts, induction of anesthesia was facilitated by 5% isoflurane and oxygen (1 L/min), then maintained at 1.5–2% isoflurane. Due to the impact of oxygen on microbubble circulation half-life^30,31^, medical air was used as a carrier gas during sonication and imaging. Hair overlaying the skull was removed with depilatory cream and a 22-gauge angiocath was placed in the tail vein. During structural magnetic resonance imaging (MRI) and sonications, animals were positioned supine on an MRI-compatible sled with the dorsal surface of the skull coupled to a degassed, deionized water-filled polyimide window with ultrasound gel. Body temperature was maintained with heated saline bags.

### Focused ultrasound and microbubble exposure

**Figure 2:**
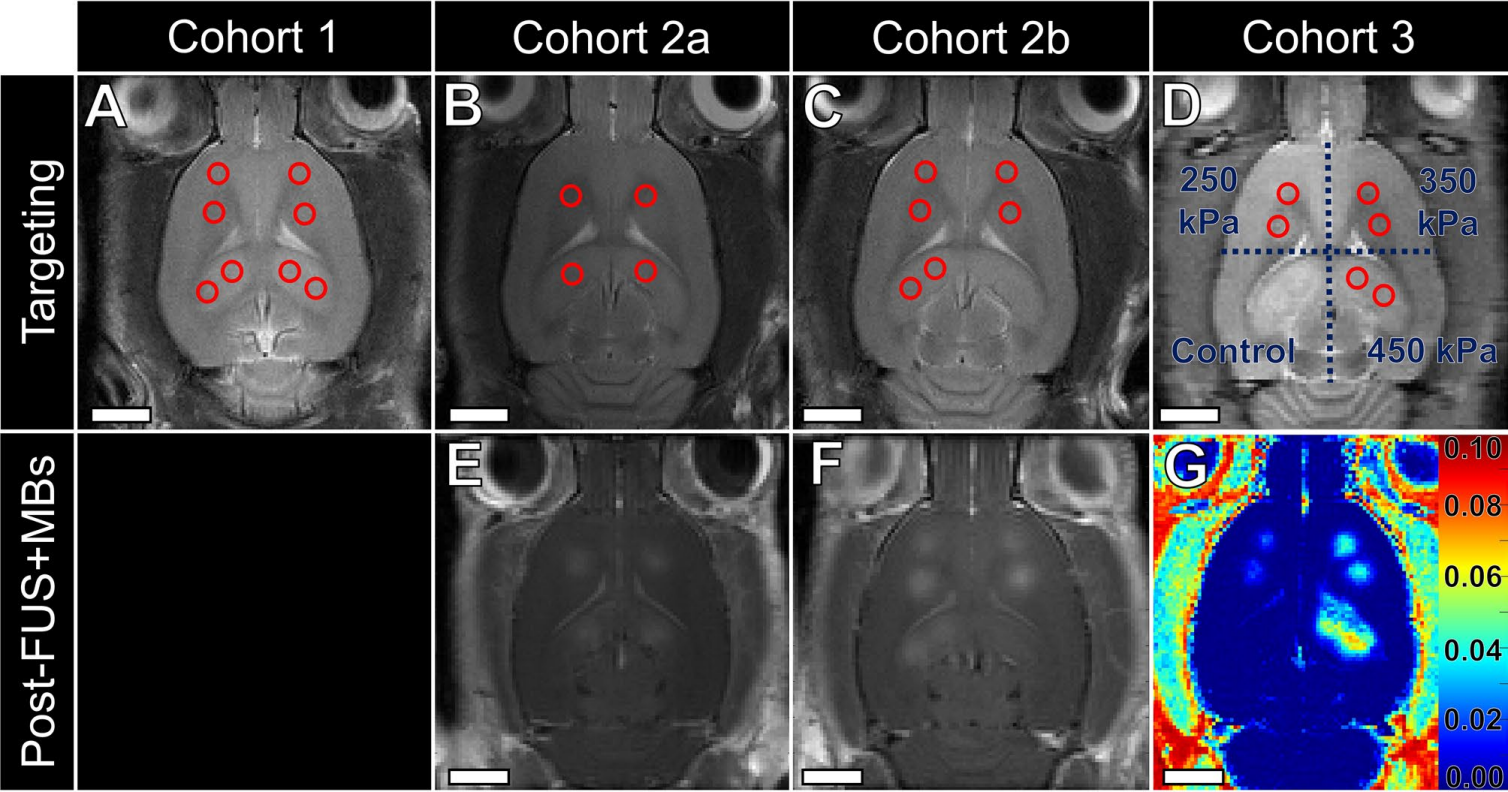
Targeting and post-sonication imaging for each animal cohort. In each animal cohort, magnetic resonance (MR) images were acquired using a T2-weighted sequence for target selection (top row). (**A**) Animals in cohort #1 each received fixed peak negative pressure (PNP; 250 kPa) sonications at 8 targets (red open circles) with no post-focused ultrasound and microbubble (FUS + MB) exposure imaging. Animals in cohort #2 each received sonications at either 4 (**B**; cohort #2a) or 6 targets (**C**; cohort #2b). PNP was calibrated at each target by acoustic feedback control. (**E**, **F**) Contrast-enhanced T1-weighted imaging was performed approximately 10 min following sonication. (**D**) Animals in cohort #3 each received fixed PNP sonications at 6 targets with (G) post-FUS + MB exposure dynamic contrast-enhanced-MRI to assess blood–brain barrier permeability (colorbar indicates K^trans^ of gadobutrol in units of min^−1^). (**D**) Two targets of equal PNP were placed in each quadrant, while the remaining quadrant served as a non-sonicated control region. The quadrant acting as the non-sonicated control region for animals in cohorts 2b and 3 was shifted to account for regional variance. Scale bars = 4 mm.

Ultrasound was delivered using a pre-clinical, MRI-guided FUS system (a prototype similar to LP100; FUS Instruments Inc., Toronto, ON, Canada), equipped with an in-house manufactured, spherically focused transducer (focal number = 0.8, external diameter = 75 mm, transmit frequency (ƒ) = 580 kHz). Pressure calibration for the transducer was performed using a planar fiber optic hydrophone (active tip diameter = 10 μm; Precision Acoustics Ltd., Dorset, UK). Transducer movement within the tank of degassed, deionized water was controlled with a motorized positioning system (3 degrees of freedom). MRI spatial coordinates were coregistered to the transducer positioning system to allow targets to be chosen from structural MR images. For all sonications, ultrasound was delivered in 10 ms bursts with acoustic emissions captured with an in-house manufactured lead zirconate titanate (PZT) hydrophone located in a 25 mm opening in the centre of the transducer. Sonication parameters for each cohort are outlined in Table 2. Target selection and post-sonication imaging are depicted in Fig. 2.

**Table 2:** FUS + MB exposure parameters for each cohort of animals.

Cohort	Frequency (kHz)	Burst length (ms)	BRF (Hz)	PNP (kPa)	Targets/animal	Microbubble Administration	Bursts/target	Duration (s)
1	580	10	0.17	250	8	Bolus	110	660
2a	580	10	1	Acoustic feedback control	4	Infusion pump	130	130
2b	580	10	0.5	Acoustic feedback control	6	Infusion pump	130	260
3	580	10	0.5	250, 350, or 450	6	Infusion pump	130	260

*BRF* burst repetition frequency.

During each burst, hydrophone signals were captured (capture length = 11 ms, sampling rate = 20 MS/s) using a 14-bit scope card (ATS460; AlazarTech, Pointe-Claire, Quebec, Canada). In each animal cohort and at each target, a minimum of ten bursts were acquired without microbubbles in circulation to establish baseline values. Fast Fourier transforms (FFT) were calculated for each burst to obtain signal spectra, from which specific frequencies of interest (e.g. 1.5*f* and 2*f*) were analyzed (integration bandwidth =  ± 0.2 kHz). Wideband emissions were monitored at 890 kHz ± 5 kHz, corresponding to the peak sensitivity of the hydrophone.

### Cohort #1: Half-life in circulation

Decay in the magnitude of second harmonic (2*f*) emissions was used to estimate the half-life in circulation for each microbubble formulation in animals from cohort #1 (n = 3 animals/microbubble formulation). Eight targets per brain were sonicated at a peak negative pressure (PNP) of 250 kPa (measured in water without skull attenuation) and burst repetition frequency (BRF) of 0.17 Hz for 11 min. Microbubbles were administered intravenously as a bolus. In each animal, the six targets displaying the greatest difference between baseline (i.e. no microbubbles in circulation) and the maximum magnitude of 2*f* emissions were selected (to remove low-signal targets) and averaged across all targets at each burst number in the series with baseline measurements subtracted. Decay in the magnitude of 2*f* emissions over time was fit to a biexponential function. Half-life in circulation was estimated in each animal during the terminal phase of the decay (i.e. following the distribution phase).

### Cohort #2: Acoustic feedback control algorithm testing

Animals in cohort #2 were used to compare the performance of a previously described^32^ acoustic feedback control algorithm between microbubble formulations (n = 7, 8, and 9 animals, for Definity, BG8774, and MSB4, respectively). Briefly, each target was sonicated at an initial PNP of 128 kPa, which was increased by 8 kPa after each burst. Microbubbles were administered intravenously at a rate of 0.5 ml/min (0.5 ml vol.) using an infusion pump (Nanojet syringe pump, Chemyx Inc., Stafford, TX, USA). During sonication, acoustic emissions at 1.5*f* and 2.5*f* were monitored; once the magnitude of emissions at either frequency passed the mean of baseline (i.e. no microbubbles in circulation) plus 10 standard deviations of the mean (aka. triggering PNP), PNP was dropped by 50% and maintained at this level for the remainder of sonication. This strategy of acoustic feedback control is designed to calibrate PNP based on in vivo microbubble response^32^ and forms the basis of the algorithm employed for transcranial FUS + MB exposures in phase I clinical trials at *Sunnybrook Research Institute*^4–6,33,34^. Gadobutrol (0.2 ml/kg; Gadovist 1.0, Bayer Inc., Toronto, ON) was administered prior to sonication to assess BBB permeability enhancement.

Animals in cohort #2 were sonicated at four (cohort #2a; n = 13) or six (cohort #2b; n = 11) targets per brain. Targeting is depicted in Fig. 2 and sonication parameters in Table 2. Magnitude of 1.5*f*, 2*f*, and wideband emissions were calculated for each target by subtracting the corresponding baseline signal (i.e. no microbubbles in circulation) values from each burst. Exposure-average magnitudes were calculated by averaging across all bursts at a given target. For all analyses, targets where the triggering PNP was two standard deviations below the mean were excluded (9/118 targets excluded); these targets were presumed to be triggered from signals outside of the brain (e.g. bubbles in ultrasound gel). Animals were sacrificed 24 h (n = 8) or 7 days (n = 16) post-FUS + MB exposure for histological assessment.

### Cohort #3: BBB permeability enhancement and relative gene expression

To compare the effects of microbubble formulation on relationships between BBB permeability enhancement, acoustic emissions, and relative gene expression, animals in cohort #3 (n = 4 animals/microbubble formulation) were each sonicated at 6 targets. Two targets, both with fixed PNPs of 250, 350, or 450 kPa, were placed in each quadrant of the brain, with one quadrant acting as a non-sonicated control region (Fig. 2D). To mitigate potential impacts of biological variance between brain regions, within each microbubble group, the quadrant acting as the non-sonicated control region was rotated between animals. This ensured that each quadrant was insonated with each of the fixed PNPs within a microbubble group. Microbubbles were administered intravenously at a rate of 0.5 ml/min (0.5 ml vol.) using an infusion pump (Nanojet syringe pump, Chemyx Inc., Stafford, TX, USA).

BBB permeability enhancement was assessed by dynamic contrast-enhanced (DCE)-MRI. Following DCE-MRI, animals were administered Evans blue dye (4% solution in saline; 2 ml/kg) and maintained under anesthesia with ketamine/xylazine until sacrifice (4 h post-FUS + MB exposure). For comparisons of acoustic emissions to the transfer constant of gadobutrol across the BBB (K^trans^; i.e. quantitative measure of BBB permeability enhancement), each target was analyzed individually. For comparisons of differential gene expression to K^trans^, tissue samples collected from the two target volumes within each brain quadrant were pooled and K^trans^ values were averaged between these targets.

### Magnetic resonance imaging

All MR imaging was performed on a 7 T horizontal bore Avance BioSpec 70/30 scanner (Bruker BioSpin, Ettlingen, Germany) with a 20 cm inner diameter gradient insert coil with maximum gradient amplitude of 668 mT/m (Bruker BioSpin, Ettlingen, Germany). Images were acquired using an 8 cm inner diameter volume coil for transmit and a quadrature rat brain coil to receive (Bruker BioSpin, Ettlingen, Germany). For FUS targeting, structural T2w images were acquired using a RARE sequence with 46.2 ms TE, 4000 ms TR, and 1.0 mm slice thickness. Animals were positioned supine for pre-sonication imaging, to allow coupling of the dorsal surface of the skull to the water tank below, and prone for post-sonication imaging, to allow the receive coil to be placed in closer proximity to the targeted brain regions.

BBB permeability enhancement for animals in cohort #2 was assessed by contrast-enhanced T1w (CE-T1w) imaging approximately 10 min following FUS + MB exposure using a RARE sequence with 10 ms TE, 500 ms TR, 6 averages, 1.0 mm slice thickness, 100 × 100 matrix size, and 24 × 24 mm field of view. Contrast enhancement at each target was normalized to a non-sonicated region and expressed as a percentage.

### Dynamic contrast enhanced magnetic resonance imaging and analysis

To assess the kinetics of gadobutrol between plasma and the extravascular-extracellular space in cohort #3 animals, quantitative MRI was performed beginning 15 min following the start of sonication, consisting of pre-contrast T1 mapping and DCE-MRI, as previously described^35^. Briefly, a single axial slice at the level of the dorsal hippocampus was selected for imaging with 100 × 100 matrix size, 24 mm × 24 mm field of view, and 1.0 mm slice thickness. T1 mapping was performed using an inversion recovery RARE sequence with 7 ms TE, 5000 ms TR, rare factor of 16, 1 average, and 5 inversion times: 125, 250, 500, 1500, and 4500 ms. DCE-MRI was performed using a FLASH sequence with 2.175 ms TE, 20 ms TR, 20° flip angle, and 3 averages, acquired at a temporal resolution of 6.0 s for 15 min. Gadobutrol (0.4 mmol/kg; Gadovist 1.0, Bayer Inc., Toronto, ON) was administered intravenously as a bolus after 1 min (10 pre-contrast images), followed by an additional 14 min of imaging.

K^trans^ of gadobutrol across the BBB was calculated from T1 mapping and DCE-MRI, as previously described^35^. Briefly, pre-contrast T1 within each region of interest or on a voxel-by-voxel basis was calculated from inversion recovery RARE images. Contrast agent concentration as a function of time was calculated based on the longitudinal relaxivity of gadobutrol (4.2 s^−1^ mM^−1^ in whole blood at 37 °C in a 7 T field^36^), the T1 of tissue as a function of time, and the pre-contrast T1 of tissue. Gadobutrol concentration was fit to a modified Tofts–Kermode model^37^ that accounts for the presence of separate intravascular and extravascular extracellular compartments. Using a reference tissue approach^38,39^, the time-dependent concentration of gadobutrol in temporal muscle was used to estimate the arterial input function based on literature values of K^trans^ and v_e_ in rat muscle^40^.

### Histology

Animals included in cohort #2 were sacrificed at 24 h (n = 8) or 7 days (n = 16) following sonication by transcardial perfusion with ice-cold saline, followed by 10% neutral buffered formalin. Brains were extracted and post-fixed in 10% neutral buffered formalin at 4 °C overnight, then paraffin embedded. Horizontal sections (5 μm thick) were collected at 250 μm intervals and hematoxylin–eosin (H&E) stained. One section per animal, estimated to be at the plane displaying maximum gadolinium contrast enhancement in T1w images, was evaluated with a 20× objective lens using brightfield microscopy. Areas of red blood cell (RBC) extravasation were identified manually by one researcher (DM) that was blinded to experimental conditions.

### Gene expression analysis

Animals in cohort #3 (n = 12) were sacrificed at 4 h following sonication by transcardial perfusion with ice-cold saline. Brains were extracted and regions of Evans blue extravasation within each quadrant, plus a non-sonicated control region, were dissected on ice. Samples were frozen on dry ice, then stored at − 80 °C until processing. RNA was isolated from frozen tissue samples using a RNeasy Mini kit (QIAGEN, Hilden, Germany) according to the manufacturer’s instructions. RNA quality and concentration were determined using 2100 Bioanalyzer (Agilent, Santa Clara, CA, USA); all samples displayed RNA integrity numbers above 7.0. The expression of 84 genes were screened with RT^2^ Profiler PCR Array Rat Endothelial Cell Biology (QIAGEN, Hilden, Germany). CFX96 Touch Real-Time PCR Detection System (Bio-Rad Laboratories Inc., Hercules, CA, USA) was used in conjunction with RT^2^ SYBR Green qPCR Master Mix (QIAGEN, Hilden, Germany) for quantitative real-time polymerase chain reaction (qRT-PCR). Relative gene expression of each transcript was determined by normalizing against the mean Ct value of 5 housekeeping genes (*Actb*, *B2m*, *Hprt1*, *Ldha*, *and Rplp1*), using the ΔΔCt method. Low expression genes (i.e. Ct values > 35) were excluded from analysis. Individual genes were excluded from analysis if 25% or more samples displayed low expression (excluded: *Agtr1b, Cxcl2, Cxcr5, Edn2, Il11, Il3, Mmp1a, Nppb, and Sell*). Within each animal, log2 fold change for each target location was calculated relative to the non-sonicated control region. Individual samples were excluded from analysis if 25% or more genes from that sample displayed low expression (5/48 samples excluded).

### Statistics

All statistical analyses were performed using R version 3.5.1. *Cohort #1:* Differences in microbubble half-life in circulation were compared between formulations by one-way analysis of variance (ANOVA) with post-hoc Tukey’s HSD test. *Cohort #2:* ANOVAs revealed no significant differences for any outcome measures between Cohorts #2a and #2b; these cohorts were pooled for all subsequent analyses. Exposure-average magnitude of 2*f* emissions, triggering PNP, and gadolinium contrast enhancement were compared between microbubble formulations by one-way ANOVAs with post-hoc Tukey’s HSD tests. The relationship between exposure-average magnitude of 2*f* emissions and gadolinium contrast enhancement between microbubble formulations was assessed by analysis of covariance (ANCOVA), allowing unequal slopes. Post-hoc, pairwise (i.e. between microbubble formulations) ANCOVAs were performed with false discovery rate (FDR) correction for multiple comparisons. Proportions of total bursts containing wideband emissions were compared between microbubble formulations by Fisher’s exact test and post-hoc chi-squared tests with FDR correction. Equality of variance in gadolinium contrast enhancement between microbubble formulations was assessed by pairwise Levene’s tests with FDR correction. *Cohort #3:* Least squares regression was used to assess the relationship between relative gene expression and K^trans^ for all samples (i.e. microbubble formulation not considered), with FDR correction. To explore the impact of microbubble formulation on relationships between K^trans^ and relative expression of inflammation-related genes, genes belonging to the GO-term ‘Inflammatory Response’ were grouped; ANCOVAs, allowing unequal slopes, were used to assess significance with FDR correction. Post-hoc, pairwise ANCOVAs (i.e. between microbubble formulations) were performed with FDR correction. For all analyses, a p-value of 0.05 was used as the threshold for statistical significance. Unless otherwise specified, variance is expressed as standard deviation of the mean.

## Results

### Microbubble half-life in circulation

**Figure 3:**
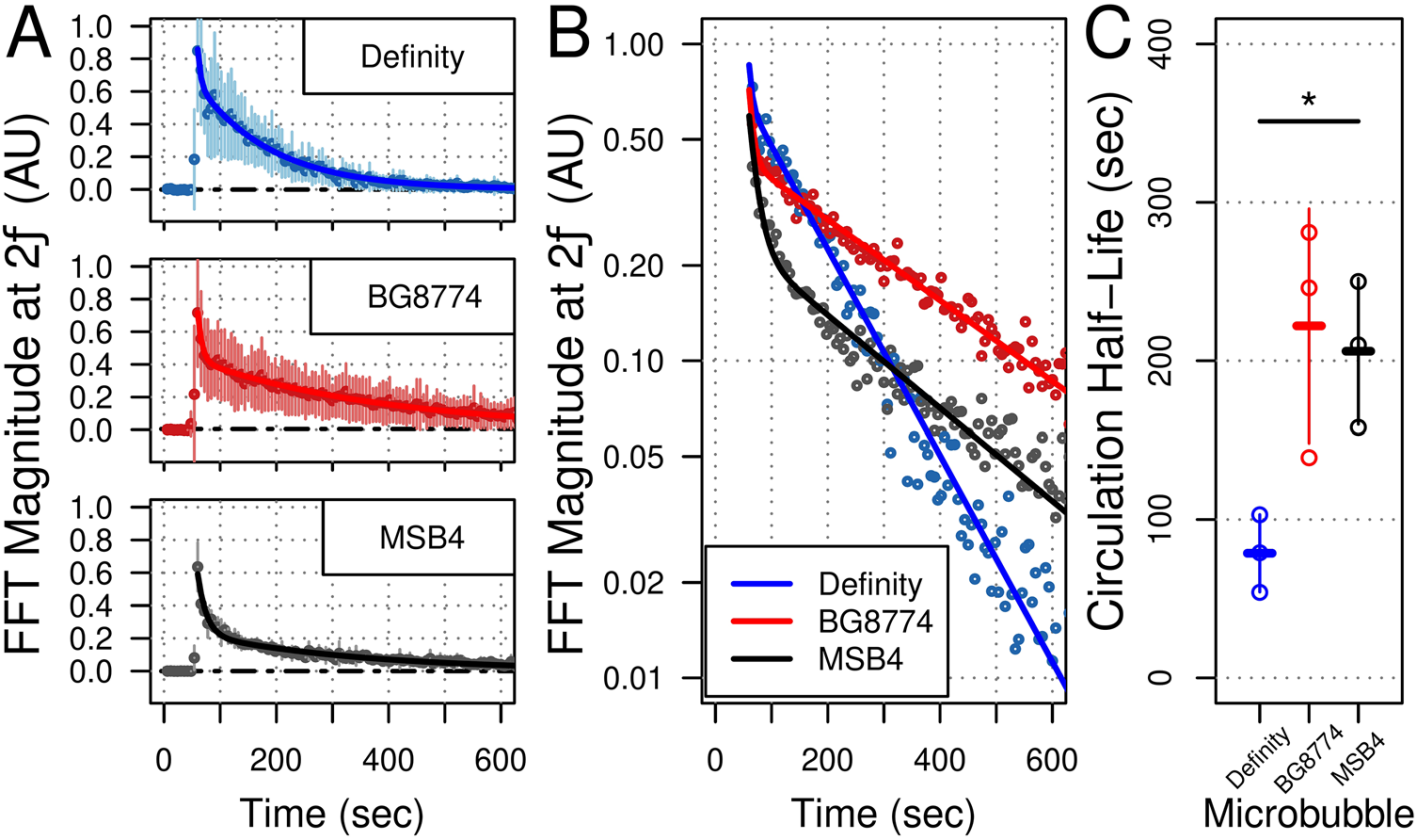
Half-life in circulation. (**A**) Decay in the magnitude of 2*f* emissions were used to estimate the concentration of each microbubble formulation as a function of time (n = 3 animals/microbubble formulation). The mean decay for each microbubble formulation is depicted. Each microbubble formulation displayed a biphasic decay; half-life in circulation was estimated during the terminal phase. (**B**) Decay in the magnitude of 2*f* emissions plotted on a semi-log scale highlight the differences in decay characteristics for each microbubble formulation. (**C**) Definity displayed a mean half-life of 79 s ± 25 s, significantly less than MSB4 at 206 s ± 46 s (p = 0.04). BG8774 displayed a mean half-life of 222 s ± 73 s, trending towards significantly greater than Definity (p = 0.06). *AU* arbitrary units, *FFT* fast Fourier transform.

Decay in the magnitude of 2*f* emissions was used to estimate the half-life of each microbubble formulation in circulation (Fig. 3). All microbubble formulations exhibited a biphasic decay in the magnitude of 2*f* emissions over time, with a rapid decay directly following bolus administration after which a more gradual decay was exhibited. The latter portion of the curve (i.e. the terminal phase) was used to estimate the circulation half-life in each cohort #1 animal. Definity, BG8774, and MSB4 displayed mean circulation half-lives of 79 s ± 25 s, 222 s ± 73 s, and 206 s ± 46 s, respectively. The mean circulation half-life of Definity was significantly less than MSB4 (p = 0.04) and trended towards significantly less than BG8774 (p = 0.06).

### Acoustic feedback control algorithm testing

**Figure 4:**
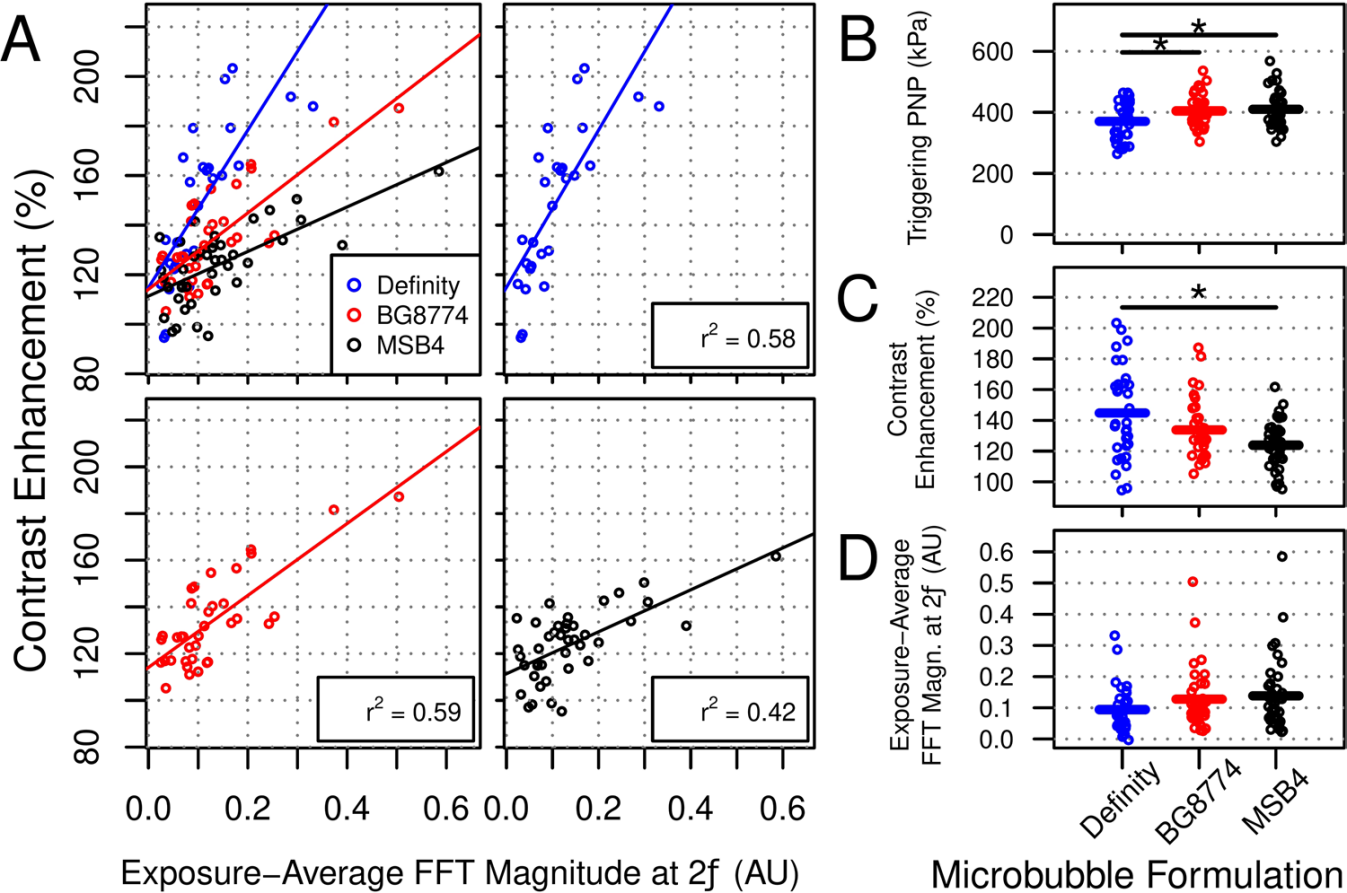
Acoustic feedback control algorithm testing. The peak negative pressures (PNPs) of sonications for animals in cohort #2 were calibrated based on the detection of ultraharmonic emissions. (**A**) Relative gadolinium contrast enhancement from contrast-enhanced T1-weighted imaging post-sonication is plotted in relation to exposure-average magnitude of 2*f* emissions for each microbubble formulation. Positive linear correlations were observed for all groups, with adjusted r^2^ values of 0.58, 0.59, and 0.42, for Definity, BG8774, and MSB4, respectively. Pairwise analyses indicated that each microbubble formulation displayed a significantly different relationship compared to the other two formulations. (**B**) The mean triggering PNPs (i.e. PNP required to detect ultraharmonic emissions) were 370 kPa ± 60 kPa, 405 kPa ± 51 kPa, and 410 kPa ± 59 kPa, for Definity, BG8774, and MSB4, respectively. Significant differences were detected between Definity and both BG8774 (p = 0.03) and MSB4 (p = 0.01). (**C**) Mean relative gadolinium contrast enhancement approximately 10 min following sonication was significantly greater at targets sonicated with Definity in circulation than MSB4 (p < 0.001). (**D**) No significant differences were detected in mean exposure-average magnitude of second harmonic emissions between microbubble formulations. *AU* arbitrary units, *FFT* fast Fourier transform.

PNP of FUS + MB exposures for animals in cohort #2 were calibrated based on the detection threshold for ultraharmonic (1.5*f*) emissions (i.e. triggering PNP). At targets sonicated with Definity, BG8774, and MSB4 in circulation, the mean thresholds for detecting 1.5*f* emissions were 370 kPa ± 60 kPa, 405 kPa ± 51 kPa, and 410 kPa ± 59 kPa, respectively (Fig. 4B). The mean triggering PNP for Definity was significantly less than for BG8774 (p = 0.03) or MSB4 (p = 0.01). The magnitude of 1.5*f* emissions at the triggering bursts were not predictive of gadolinium contrast enhancement (Supplementary Fig. 1). The acoustic feedback control algorithm employed was effective in minimizing wideband emissions, with 1% or fewer bursts displaying evidence of inertial cavitation for sonications with Definity (25/4680), BG8774 (52/5200), or MSB4 (25/5460) in circulation (Supplementary Fig. 2); however, sonications with BG8774 in circulation contained a significantly greater proportion of bursts with wideband emissions than Definity (p = 0.02) or MSB4 (p = 0.005). The magnitude of maximum wideband emissions per target were not predictive of gadolinium contrast enhancement (Supplementary Fig. 3).

BBB permeability enhancement was assessed by CE-T1w imaging approximately 10 min following FUS + MB exposure for animals in cohort #2 (Fig. 4C). Mean gadolinium contrast enhancement relative to a non-sonicated control region was 145% ± 30%, 134% ± 19%, and 124% ± 15% at targets sonicated with Definity, BG8774, and MSB4 in circulation, respectively; a significant difference was detected between Definity and MSB4 (p < 0.001). The variance in gadolinium contrast enhancement at targets sonicated with Definity in circulation was significantly greater than with BG8774 (p < 0.01) or MSB4 (p < 0.01). No significant differences were detected in mean exposure-average magnitude of 2*f* emissions between microbubble formulations (Fig. 4D).

Potential effects of microbubble formulation on the relationship between exposure-average magnitude of 2*f* emissions and gadolinium contrast enhancement were assessed in cohort #2 animals (Fig. 4A). For targets sonicated in the presence of Definity, BG8774, and MSB4, adjusted r^2^ values of 0.58, 0.59, and 0.42 were observed, respectively. ANCOVA revealed a significant effect of microbubble formulation on this relationship (p < 0.001). Post-hoc tests detected significant differences between all pairwise comparisons of microbubble formulations (p < 0.05).

**Figure 5:**
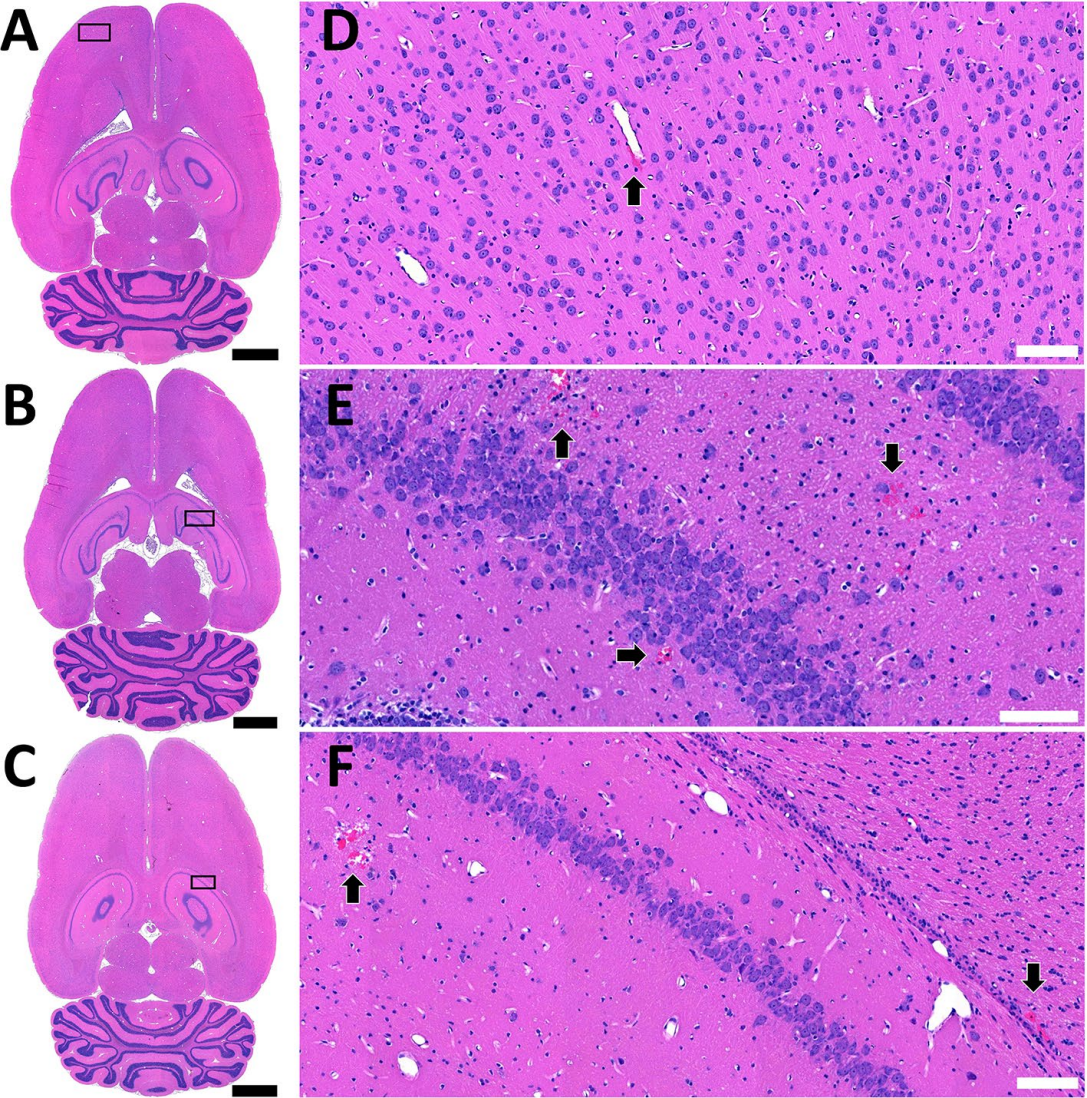
Red blood cell extravasation. Overt tissue damage in cohort #2 animals was assessed at 24 h and 7 days following sonication in H&E stained sections. No evidence of red blood cell (RBC) extravasation was evident at 7 days for any sonications. The largest regions of RBC extravasation 24 h following focused ultrasound and microbubble exposure (cohort #2a) are depicted for sonications with (**A**, **D**) Definity, (**B**, **E**) BG8774, or (**C**, **F**) MSB4 in circulation. White arrows highlight regions of RBC extravasation. Black scale bars = 2 mm. White scale bars = 100 μm.

The impact of FUS + MB exposure on RBC extravasation was assessed in H&E stained sections from animals in cohort #2, sacrificed at 24 h (n = 8) and 7 days (n = 16) following sonication. For all microbubble formulations, no regions of RBC extravasation were observed 7 days post-FUS + MB exposure (86 targets total). At 24 h, small regions of RBC extravasation (all < 300 μm in diameter) were observed in less than 50% of the targeted regions for all microbubble formulations. Brightfield images demonstrating the largest regions of RBC extravasation for each microbubble formulation are displayed in Fig. 5.

### Acoustic emissions and K^trans^ for sonications at fixed peak negative pressure

**Figure 6:**
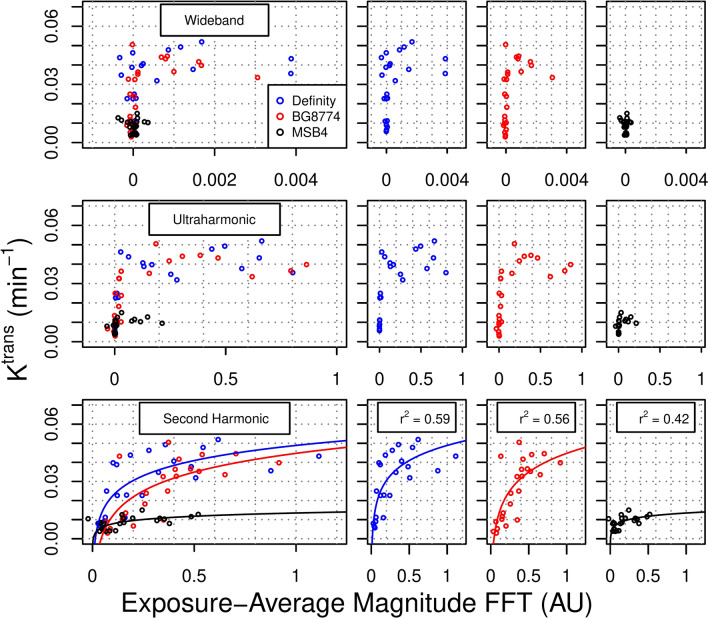
Relationship between acoustic emissions and K^trans^. Animals in cohort #3 were sonicated at a range of fixed peak negative pressures. Dynamic contrast-enhanced magnetic resonance imaging was used to quantitatively assess blood–brain barrier permeability to gadobutrol (K^trans^). Exposure-average magnitudes of ultraharmonic and wideband emissions for both Definity and BG8774 were negligible at targets displaying K^trans^ below 0.03 min^−1^. All microbubble formulations exhibited a semi-log relationship between exposure-average magnitudes of 2*f* emissions and K^trans^, with adjusted r^2^ values of 0.59, 0.56, and 0.42, for Definity, BG8774, and MSB4, respectively. *AU* arbitrary units, *FFT* fast Fourier transform.

As a quantitative measure of BBB permeability following FUS + MB exposures at fixed PNPs, K^trans^ was estimated 15 min following the start of sonication from data collected using DCE-MRI for animals in cohort #3. For Definity, BG8774, and MSB4, adjusted r^2^ values for the correlations between exposure-average magnitude of 2*f* emissions (log-transformed) and K^trans^ were 0.59, 0.56, and 0.42, respectively (Fig. 6). ANCOVA revealed a significant effect of microbubble formulation on this relationship (p < 0.001). Post-hoc tests detected significant differences between MSB4 and both Definity (p < 0.001) and BG8774 (p < 0.001), but not between Definity and BG8774 (p = 0.4). For fixed PNPs of 350 and 450 kPa, mean K^trans^ at targets sonicated with either Definity (p < 0.05) or BG8774 (p < 0.05) in circulation was significantly greater than at targets sonicated with MSB4 in circulation (Supplementary Fig. 4).

Evaluation of wideband emissions indicated that for FUS + MB exposures with Definity or BG8774 in circulation, sustained inertial cavitation was detectable at a portion of targets which displayed K^trans^ values above 0.03 min^−1^ (Fig. 6); exposure-average magnitude of wideband emissions were significantly less at targets sonicated with MSB4 in circulation, compared to either Definity (p < 0.05) or BG8774 (p < 0.05).

### K^trans^ and relative gene expression for sonications at fixed peak negative pressure

Gene expression in tissue collected 4 h following sonications at fixed PNPs were compared to K^trans^ at those targeted locations for animals in cohort #3. For several genes, the variance in relative expression was, in part, explained by the magnitude of K^trans^ when microbubble formulation was not considered (i.e. all samples pooled; Table 3); significant correlations were detected between K^trans^ and relative expression of *Ccl2*, *Cxcl1*, *Il6*, *Serpine1*, and *Selp*, which all exhibited adjusted r^2^ values above 0.3 and positive slopes at targets sonicated with Definity, BG8774, or MSB4 in circulation. When only targets sonicated with Definity or BG8774 were pooled (Table 4), significant correlations were detected between K^trans^ and relative expression of *Ccl2*, *Cxcl1*, *Icam1*, *Il1b*, *Il6, Serpine1*, and *Tnf*, which all exhibited adjusted r^2^ values above 0.5 and positive slopes.

**Table 3:** Genes displaying significant correlations between relative expression and K^trans^ across all microbubble formulations.

Genes	Adjusted p-value	Adjusted r^2^	Slope	Intercept
*Ccl2*	< 0.001	0.34	114.40	1.07
*Cxcl1*	< 0.001	0.41	106.28	0.61
*Fas*	< 0.001	0.25	32.39	0.17
*Icam1*	< 0.001	0.25	52.98	0.38
*Il1b*	< 0.001	0.26	78.15	0.94
*Il6*	< 0.001	0.38	57.16	− 0.15
*Itga5*	< 0.001	0.26	45.98	0.28
*Serpine1*	< 0.001	0.32	79.71	1.00
*Selp*	0.008	0.35	76.52	0.71
*Timp1*	0.008	0.21	38.03	0.37
*Tnf*	0.022	0.18	58.17	0.88
*Hmox1*	0.027	0.17	39.56	0.08

Units of slope are log2 fold change in relative gene expression/min^−1^ (K^trans^ of gadobutrol).

**Table 4:** Genes displaying significant correlations between relative expression and K^trans^ across targets sonicated with Definity or BG8774 in circulation.

Genes	Adjusted p-value	Adjusted r^2^	Slope	Intercept
*Ccl2*	< 0.001	0.63	134.34	− 0.10
*Cxcl1*	< 0.001	0.73	121.23	− 0.29
*Icam1*	< 0.001	0.50	62.09	− 0.17
*Il1b*	< 0.001	0.63	94.73	− 0.03
*Il6*	< 0.001	0.58	61.88	− 0.42
*Itga5*	< 0.001	0.42	52.20	− 0.04
*Serpine1*	< 0.001	0.53	90.08	0.31
*Timp1*	< 0.001	0.44	45.23	− 0.03
*Tnf*	< 0.001	0.55	81.03	− 0.30
*Fas*	0.008	0.33	34.34	0.05
*Selp*	0.015	0.45	78.58	0.40
*Hmox1*	0.040	0.23	47.80	− 0.37

Units of slope are log2 fold change in relative gene expression/min-1 (K^trans^ of gadobutrol).

**Figure 7:**
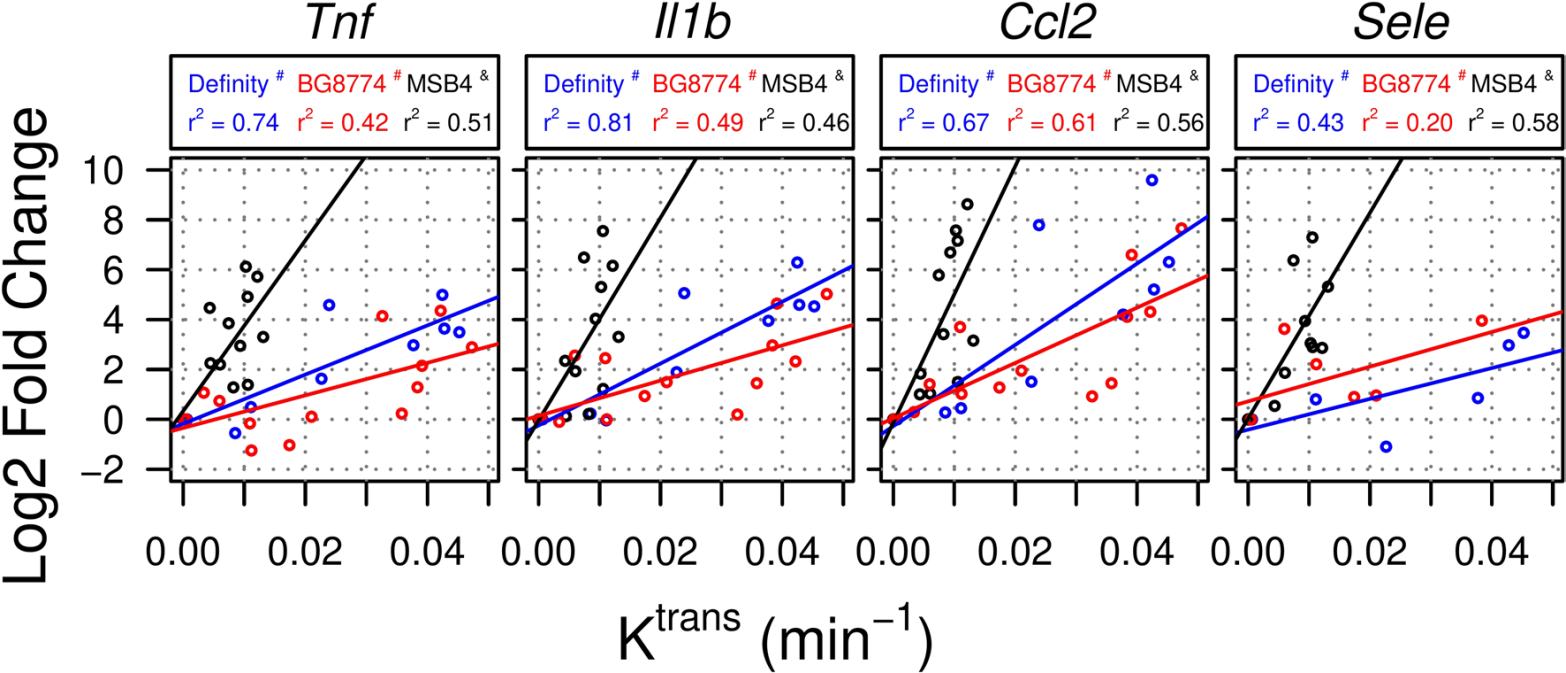
Relationship between K^trans^ and relative gene expression for inflammatory mediators significantly influenced by microbubble formulation. For sonications with each microbubble formulation, significant correlations were detected between K^trans^ and relative expression of *Tnf*, *Il1b*, *Ccl2*, and *Sele*. These relationships were significantly different between sonications with MSB4 in circulation, compared to either Definity or BG8774. Disparate symbols (# vs &) indicates significantly different slopes (K^trans^ vs relative gene expression) in pairwise post-hoc comparisons between microbubble formulations.

When considering microbubble formulation in circulation during FUS + MB exposure, the relationship between relative expression and K^trans^ was significantly affected for several genes involved in inflammatory processes (GO term ‘Inflammatory Response’; Table 5). ANCOVAs detected significantly different relationships between K^trans^ and relative expression of *Tnf*, *Ccl2*, *Il1b*, and *Sele* when microbubble formulation in circulation was considered (Fig. 7). Post-hoc pairwise comparisons revealed that this relationship was significantly different between Definity or BG8774 and MSB4 for relative expression of *Tnf*, *Ccl2*, *Il1b*, and *Sele*.

**Table 5:** Correlations between K^trans^ and relative gene expression (GO term ‘Inflammatory Response’) for each microbubble formulation.

Genes	Adjusted p-value (ANCOVA)	Definity			BG8774			MSB4		
		Adjusted r^2^	Slope	Intercept	Adjusted r^2^	Slope	Intercept	Adjusted r^2^	Slope	Intercept
*Ccl2*	0.021	0.67	162.87^#^	− 0.27	0.61	110.81^#^	0.05	0.56	515.54^&^	− 0.14
*Il1b*	0.021	0.81	124.10^#^	− 0.24	0.49	70.72^#^	0.14	0.46	406.63^&^	− 0.04
*Sele*	0.021	0.43	61.75^#^	− 0.41	0.20	69.60^#^	0.72	0.58	398.72^&^	0.05
*Tnf*	0.021	0.74	98.61^#^	− 0.17	0.42	65.30^#^	− 0.34	0.51	334.88^&^	0.40
*Cxcl1*	0.147	0.86	136.79	− 0.53	0.61	109.20	− 0.13	0.46	397.22	− 0.29
*Selp*	0.147	0.82	82.55	− 0.03	0.29	78.78	0.63	0.63	349.11	− 0.53
*Tgfb1*	0.209	0.80	29.02	− 0.16	− 0.04	7.68	− 0.09	0.37	65.63	0.00
*Thbs1*	0.474	0.50	53.44	− 0.14	− 0.04	10.27	0.33	0.00	88.63	0.17
*Calca*	0.624	− 0.11	− 4.73	− 0.48	− 0.04	11.64	0.04	0.02	− 82.16	− 0.27
*F2r*	0.624	− 0.05	− 3.37	0.15	0.14	− 18.09	0.24	− 0.07	9.92	0.12
*Il6*	0.624	0.70	67.99	− 0.47	0.47	56.92	− 0.37	0.13	141.09	− 0.39
*Itgav*	0.624	− 0.08	− 4.18	0.20	− 0.07	− 3.36	0.32	0.21	59.07	− 0.02
*Pf4*	0.624	0.35	26.65	0.15	− 0.07	2.72	0.55	− 0.01	64.6	− 0.04
*Ptgs2*	0.840	0.21	28.10	0.01	0.11	42.87	− 0.33	0.05	94.17	− 0.01
*Ccl5*	0.921	0.02	30.18	− 0.77	0.00	22.58	0.24	− 0.04	74.38	0.01
*Kit*	0.921	0.22	− 19.50	− 0.20	− 0.06	− 8.10	− 0.27	− 0.01	− 44.31	0.04
*Cx3cl1*	0.994	− 0.09	− 4.710	0.25	− 0.06	− 3.92	0.14	− 0.08	0.43	0.00

Disparate symbols (# and &) indicate significantly different slopes (K^trans^ vs relative gene expression) in pairwise post-hoc comparisons between microbubble formulations. Units of slope are log2 fold change in relative gene expression/min^−1^ (K^trans^ of gadobutrol).

*ANCOVA* analysis of covariance.

### Within-sample correlations in relative gene expression

Correlations in relative expression between gene pairs within samples were assessed across all experimental conditions. A heatmap of within-sample gene–gene correlations in relative expression is shown in Supplementary Fig. 5. Gene pairs which showed strong correlations in relative expression within samples, included *Il1b-Ccl2*, *Cxcl1-Ccl2*, *Icam1-Ccl2*, *Serpine1-Cxcl1*, *Icam1-Cxcl1*, *Il1b-Cxcl1*, *Icam1-Il1b*, and *Tnf-Il1b*, which displayed r^2^ values of 0.86, 0.78, 0.73, 0.74, 0.71, 0.77, 0.60, and 0.65, respectively (Supplementary Fig. 6).

## Discussion

The present study investigated the impacts of FUS + MB exposure on BBB permeability enhancement, RBC extravasation, and the transcription of inflammatory mediators, comparing these outcome measures between sonications employing different microbubble formulations. To the best of our knowledge, this is the first study to directly compare the effects of FUS + MB exposure in the brain between sonications employing monodisperse and polydisperse microbubbles. Results indicate that acute biological responses to sonication are not solely dependent on the initial magnitude of BBB permeability enhancement induced; microbubble characteristics (i.e. size distribution, gas and shell compositions) may also influence these responses. Additionally, these results emphasize the notion that when predicting biological responses based on acoustic emissions or when employing acoustic feedback control strategies that calibrate PNP based on in vivo microbubble responses, it may be necessary to consider microbubble characteristics.

### Acoustic emissions and K^trans^ for sonications at fixed peak negative pressure

To examine the wide spectrum of biological responses that can be induced by FUS + MB exposures, from BBB permeability enhancement suitable for drug delivery to extensive tissue damage, animals in cohort #3 were sonicated with a range of fixed PNPs (i.e. 250, 350, 450 kPa). When employing high fixed PNP, K^trans^ plateaued at approximately the level observed in temporal muscle for exposures with Definity or BG8774 in circulation. At a subset of these targets, acoustic emissions indicated the presence of substantial inertial cavitation, which has been shown to damage the endothelial cells lining cerebrovasculature^41^. This effect—a plateau in the K^trans^ of an MRI contrast agent—has previously been observed when employing high PNPs^20^ and is likely indicative of gadobutrol extravasation driven in large part by microhemorrhage.

The relationship between exposure-average magnitude of 2*f* emissions and K^trans^ at targets sonicated with Definity or BG8774 in circulation displayed semi-log relationships. This result builds on findings of previous studies which have noted linear correlations between harmonic emissions and gadolinium contrast enhancement^35,41^, but where extensive hemorrhage may not have contributed substantially to BBB permeability enhancement. At targets sonicated with MSB4 in circulation, the relationship between exposure-average magnitude of 2*f* emissions and K^trans^ was significantly different than for Definity or BG8774, with the same level of 2*f* emissions generally correlated to a lower magnitude of K^trans^ for sonications with MSB4 in circulation.

A possible explanation for these observations may involve FUS + MB-induced blood flow changes in the targeted tissue. Vasoconstriction and the transient cessation/reduction of blood flow in cerebrovasculature has been observed in vivo following FUS + MB exposures with Optison^42^ and Definity^43^. This change in vascular tone is thought to be driven by the mechanical stimulation of smooth muscle cells by oscillating microbubbles, as similar responses are observed following physical contact to arterial walls by guide wires or catheters during interventional radiology procedures^44^. The larger initial size of most MSB4 microbubbles may cause a greater degree of blood vessel wall distension or stimulation during peak microbubble expansion, which may in turn induce more severe or prolonged vasoconstriction. Vasomotor responses may also act to reduce microbubble replenishment and/or gadobutrol flow within the targeted tissue, both of which may contribute to the lower K^trans^ values observed at locations sonicated with MSB4 in circulation.

### Inflammatory response

Differences in blood flow following FUS + MB exposures may also provide an explanation for disparate relationships between K^trans^ and relative gene expression between microbubble formulations for animals in cohort #3. Targets sonicated with Definity or BG8774 in circulation displayed similar changes in relative gene expression as a function of K^trans^. When considering targets sonicated with either of these two microbubble formulations in circulation, strong correlations between K^trans^ and relative gene expression were observed for *Cxcl1*, *Ccl2*, *Il1b*, *Il6*, *Tnf*, and *Icam1* (Table 4); these correlations were weakened by the pooled analysis of all microbubble formulations (Table 3). For targets sonicated with MSB4 in circulation, the relationships between K^trans^ and the relative expression of several genes involved in acute inflammation (*Tnf*, *Ccl2*, *Il1b*, and *Sele*) were significantly different compared to Definity or BG8774. The increased relative transcription of these genes as a function of K^trans^ at targets sonicated with MSB4 in circulation suggests that acute inflammatory processes are influenced by factors beyond initial BBB permeability enhancement.

Reduced blood flow and mild hypoxia are known to exacerbate acute inflammatory responses in the brain. For example, 30 min of mild hypoxia following traumatic brain injury has been shown to increase the relative expression of proinflammatory cytokines like TNFɑ^45^, IL6^45,46^ and IL1β^45^, as well as increase astrocyte activation^47^ within 24 h of injury versus traumatic brain injury alone. Similarly, Brochu et al*.* demonstrated that the combination of hypoxia and systemic lipopolysaccharide administration, a potent stimulator of neuroinflammation, induced a greater upregulation in IL1β and MCP1 (i.e. the protein encoded by *Ccl2*) protein expression at 4 and 48 h following insult compared to either hypoxia or lipopolysaccharide administration alone^48^. It is possible that any hypothetical reduction in blood flow or mild hypoxia induced by sonications with MSB4 in circulation would exacerbate the production of inflammatory mediators following FUS + MB exposure; however it is important to emphasize that this study does not provide direct evidence of reduced blood flow or mild hypoxia during, or following, sonication.

Depending on the purpose of sonication, elevating the production of proinflammatory cytokines and chemokines without inducing overt tissue damage (i.e. RBC extravasation) may serve a therapeutic purpose. For example, this regime of FUS + MB exposure may be more conducive to amyloid beta clearance^49^, neurogenesis^50^, angiogenesis^51^, immune cell delivery^52,53^, or other therapeutic applications with potential links to neuroinflammation; however, for most drug delivery applications, efforts to minimize FUS + MB-induced inflammation, such as post-sonication administration of dexamethasone^35^ or limiting the magnitude of initial BBB permeability enhancement^26^, may be advisable. Ultimately, when discussing inflammation following FUS + MB exposures, it is important to emphasize that the magnitude of the acute response is related to, but not solely determined by, the initial magnitude of BBB permeability enhancement. This notion is a critical consideration for clinical cost benefit analyses and reinforces the necessity of employing thoroughly validated acoustic feedback control strategies, as well as continuing to improve these strategies.

### Acoustic feedback control algorithm testing

For all microbubble formulations, when PNP was calibrated based on the detection of 1.5*f* emissions (i.e. cohort #2), microhemorrhage was largely avoided, with only sparse, small regions of RBC extravasation identified at 24 h, and no signs of microhemorrhage apparent at 7 days, following FUS + MB exposure. Given the small size and transient nature of the observed RBC extravasations, the acoustic control algorithm employed here was effective in minimizing vascular damage for sonications with all microbubble formulations tested. As with experiments in cohort #3, the relationship between exposure-average 2*f* emissions and BBB permeability enhancement was influenced by microbubble formulation. The PNP threshold for detecting 1.5*f* emissions (triggering PNP) was significantly lower for targets sonicated with Definity in circulation compared to either BG8774 or MSB4, but lead to significantly greater mean gadolinium contrast enhancement than with MSB4; however, the variance in gadolinium contrast enhancement was significantly less at targets sonicated with BG8774 or MSB4 in circulation compared to Definity. Given this larger variance and the steeper slope between exposure-average 2*f* emissions and gadolinium contrast enhancement (Fig. 4A), finer control over microbubble activity may be required to improve the consistency of BBB permeability enhancement generated with Definity relative to the other microbubble formulations.

The acoustic feedback control strategy employed here was also effective in minimizing wideband emission, indicative of inertial cavitation, for sonications with all microbubble formulations tested; however, a greater proportion of total bursts displayed wideband emissions for sonications with BG8774 in circulation than the other two microbubble formulations. This may suggest that there is a more narrow range between PNPs that generate 1.5*f* emissions and those that induce inertial cavitation for BG8774, perhaps necessitating smaller step sizes during the ramp-phase of PNP calibration. Results from cohort #2 reiterate the idea that microbubble characteristics (i.e. size distribution and composition) influence their response to insonation, even when dosed by gas volume.

Previous work has demonstrated that when dosed by microbubble count, those measuring 1–2 μm in diameter induce a smaller increase in K^trans^ than 4–5 μm or 6–8 μm microbubbles^20^. Similarly, when comparing Definity to an in-house made polydisperse microbubble (dosed by microbubble count), Wang et al*.* found that K^trans^ was significantly lower at targets sonicated with Definity in circulation^21^. This may be due to a greater proportion of gas volume occupied by microbubbles larger than 4 μm in diameter for the in-house made microbubble compared to Definity. Conversely, when dosed by microbubble gas volume, Song et al. found that the fluorescence of extravasated Evans blue dye in tissue sections was approximately equal for sonications in which monodisperse microbubbles of either 2 μm or 6 μm in diameter were administered. This pattern was found to be consistent across a range of microbubble gas volumes^22^; however, it is worth noting that the sonication parameters used in that work are dissimilar in a number of ways from parameters employed in the current study (i.e. BRF, sonication duration, burst length). Results from cohort #3, comparing acoustic emissions to K^trans^, would suggest that gas volume may also be a useful guideline to normalize dose for polydisperse microbubbles of similar size distributions, but may be insufficient when size distributions are substantially different (i.e. Definity or BG8774 vs MSB4).

## Limitations

There are several limitations to the current study that authors would like to acknowledge. First, differences in shell and gas compositions for the three microbubble formulations make it difficult to discern the absolute impact of size distribution relative to microbubble composition on outcome measures; however, given the similarity in size distribution between Definity and BG8774, as well as the similarity in composition between BG8774 and MSB4, it is reasonable to conclude that size distribution had a substantial influence on the relationship between K^trans^ and the relative expression of inflammation-related genes. Similarly, it is reasonable to conclude that both microbubble composition and size distribution have a substantial influence on the relationship between PNP and the generation of acoustic emissions. Second, changes in relative gene expression were assessed at a single, early, time point following FUS + MB exposure. As such, it is difficult to make conclusions regarding the peak magnitude or duration of responses observed. While more detailed evaluations of the development and resolution of transcriptional changes have value, tissue collection at four hours following FUS + MB exposure was chosen to ensure sufficient time for changes in the expression of inflammation-related genes to manifest, based on previous literature^26,54,55^. It is worth noting that the relationships observed here between the initial magnitude of BBB permeability enhancement and the relative expression of inflammation-related genes are consistent with previous work evaluating tissue at 6 h following FUS + MB exposure^26^. Third, the FUS + MB parameters and acoustic feedback control algorithm used here were developed using Definity, hence these conditions may not be optimal for all microbubble formulations used in this study. It may be possible to develop tailor-made parameters and control strategies for each microbubble formulation that optimize safety and efficacy for BBB permeability enhancement.

## Conclusion

While much work has demonstrated the feasibility and therapeutic potential of FUS + MB-mediated BBB permeability enhancement, less investigative attention has focused on characterising the relationships between specific FUS + MB parameters and biological responses beyond vascular permeability and overt tissue damage. The work presented here demonstrates that while the initial magnitude of FUS + MB-mediated BBB permeability enhancement has clear influences on the subsequent transcription of proinflammatory cytokines, microbubble characteristics may influence these relationships and must also be considered. Additionally, this work highlights the importance of considering microbubble characteristics when designing control strategies based on acoustic emissions.

## Supplementary Information

Supplementary Figures.

## Abbreviations

ANOVA: Analysis of variance
ANCOVA: Analysis of covariance
AU: Arbitrary units
BBB: Blood–brain barrier
BRF: Burst repetition frequency
CE-T1w: Contrast-enhanced T1-weighted
DCE: Dynamic contrast-enhanced
Dn: Mean microbubble diameter
FDR: False discovery rate
FFT: Fast Fourier transform
FUS: Focused ultrasound
GSD: Geometric standard deviation
H&E: Hematoxylin–eosin
K^trans^: Transfer constant of gadobutrol
MB: Microbubble
MRI: Magnetic resonance imaging
PNP: Peak negative pressure
PZT: Lead zirconate titanate
RBC: Red blood cell
T2w: T2-weighted
χ: Shell stiffness
κ_s_: Shell viscosity
ƒ: Transmit frequency
