# Supplementary material for: Microbubble formulation influences inflammatory response to focused ultrasound exposure in the brain
Source: Sci Rep. 2020 Dec 9;10:21534. doi: 10.1038/s41598-020-78657-9 (PMC7725832; doi:10.1038/s41598-020-78657-9)
Supplement: Supplementary file 1 — Supplementary Figures. [file 41598_2020_78657_MOESM1_ESM.docx]

# Microbubble formulation influences inflammatory response to focused ultrasound exposure in the brain

Dallan McMahon (PhD) ^1,2,^ *, Anne Lassus (MSc) ^3^, Emmanuel Gaud (MSc) ^3^, Victor Jeannot (PhD) ^3^, and Kullervo Hynynen (PhD) ^1,2,4^

^1^ Physical Science Platform, Sunnybrook Research Institute, Toronto, Canada

^2^ Department of Medical Biophysics, University of Toronto, Toronto, Canada

^3^ Bracco Suisse S.A., Plan-les-Ouates, Switzerland

^4^ Institute of Biomaterials and Biomedical Engineering, University of Toronto, Toronto, Canada

* Corresponding Author ([dmcmahon@sri.utoronto.ca](mailto:dmcmahon@sri.utoronto.ca))

**SUPPLEMENTARY FIGURES**


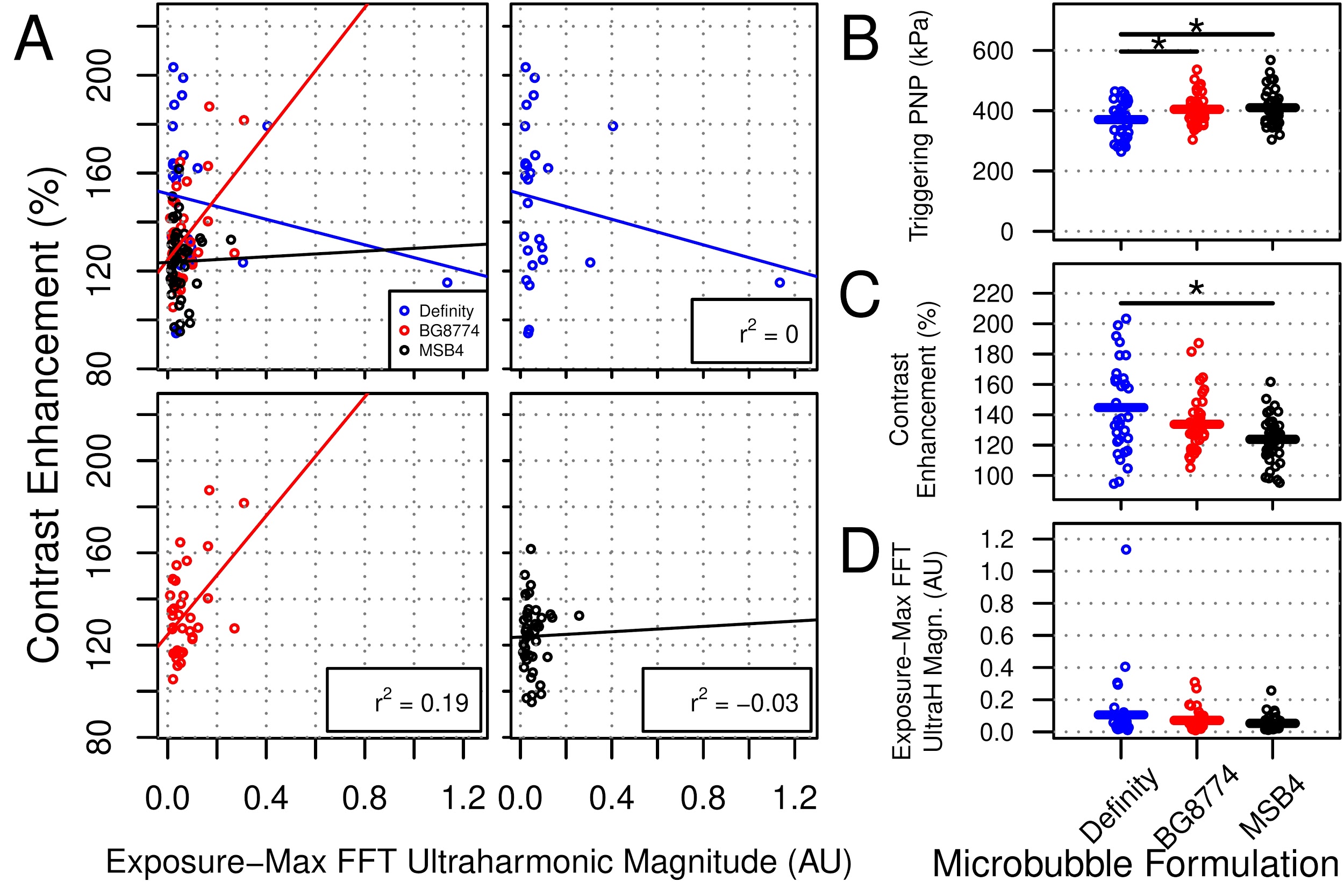


**Supplementary Figure 1: Ultraharmonic emissions during acoustic feedback control algorithm testing.** The peak negative pressure (PNP) of sonications for animals in cohort #2 were calibrated based on the detection of ultraharmonic emissions. (A) Relative gadolinium contrast enhancement from T1-weighted imaging post-sonication is plotted in relation to the magnitude of ultraharmonic emissions during the triggering burst for each microbubble formulation; clear correlations were not evident for all groups, with adjusted r^2^ values of 0.00, 0.19, and -0.03, for Definity, BG8774, and MSB4, respectively. (D) No significant differences were detected in mean magnitude of ultraharmonic emissions during the triggering burst between microbubble formulations. AU = arbitrary units, FFT = fast Fourier transform.


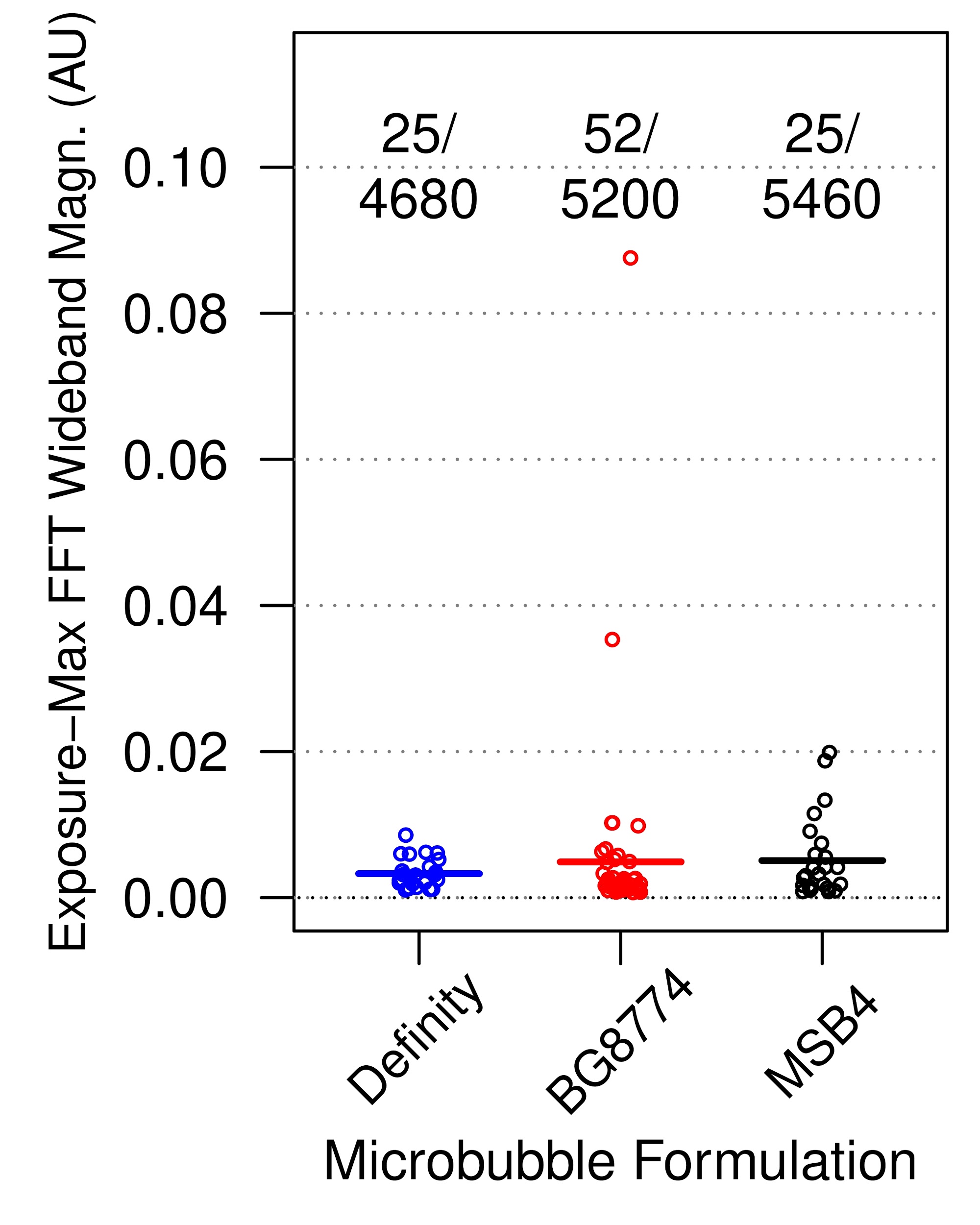


**Supplementary Figure 2: Magnitude of wideband emissions.** The peak negative pressure (PNP) of sonications for animals in cohort #2 were calibrated based on the detection of ultraharmonic emissions. For bursts displaying wideband emissions (102/15340), the magnitudes are displayed for each microbubble formulation. The proportion of bursts displaying wideband emissions was significantly greater for BG8774, compared to Definity (p = 0.02) or MSB4 (p = 0.005). AU = arbitrary units, FFT = fast Fourier transform.


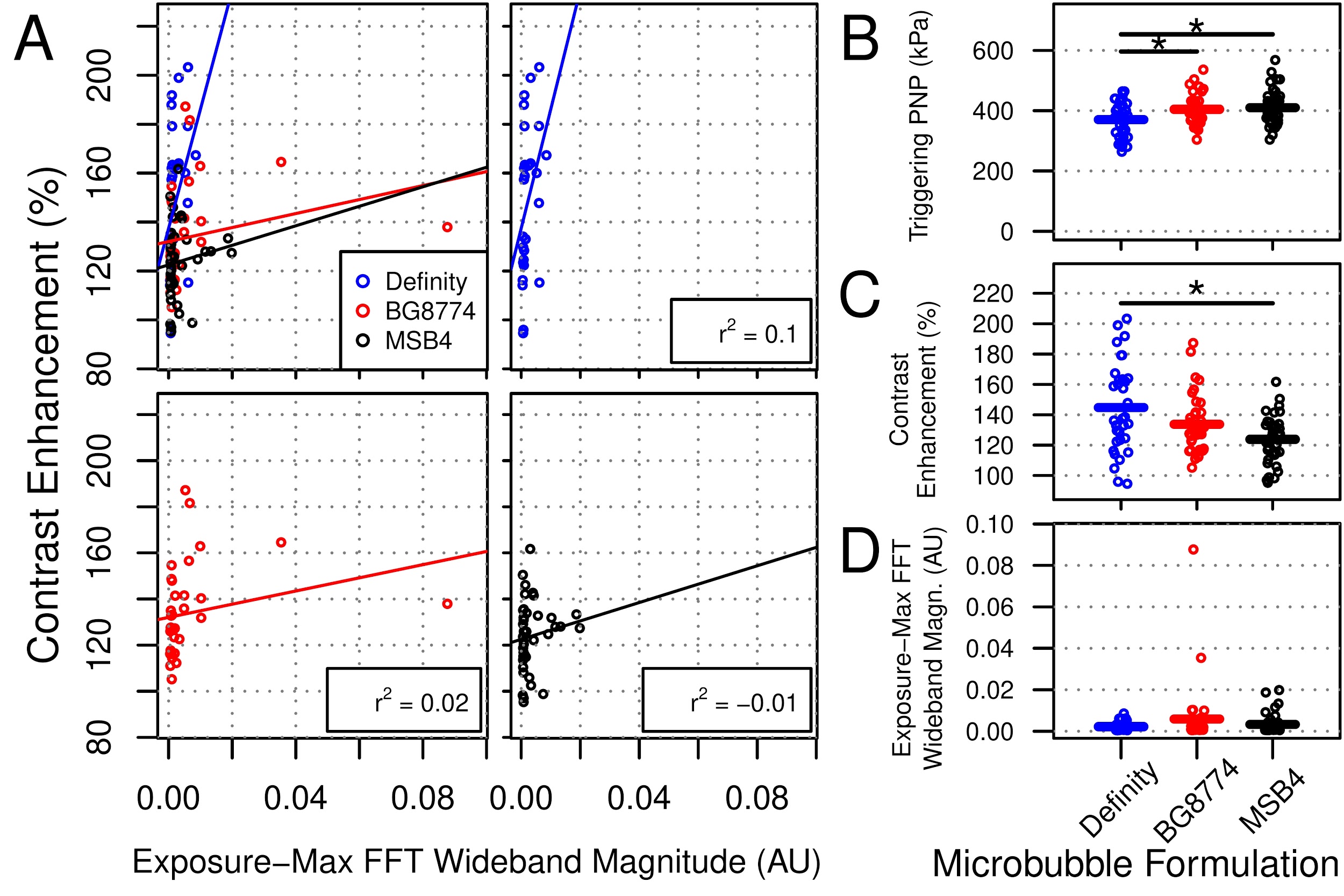


**Supplementary Figure 3: Wideband emissions during acoustic feedback control algorithm testing.** The peak negative pressure (PNP) of sonications for animals in cohort #2 were calibrated based on the detection of ultraharmonic emissions. (A) Relative gadolinium contrast enhancement from T1-weighted imaging post-sonication is plotted in relation to the magnitude of exposure-maximum wideband emissions for each microbubble formulation; clear correlations were not evident for all groups, with adjusted r^2^ values of 0.10, 0.02, and -0.01, for Definity, BG8774, and MSB4, respectively. (D) No significant differences were detected in mean exposure-maximum magnitude of wideband emissions between microbubble formulations. AU = arbitrary units, FFT = fast Fourier transform.


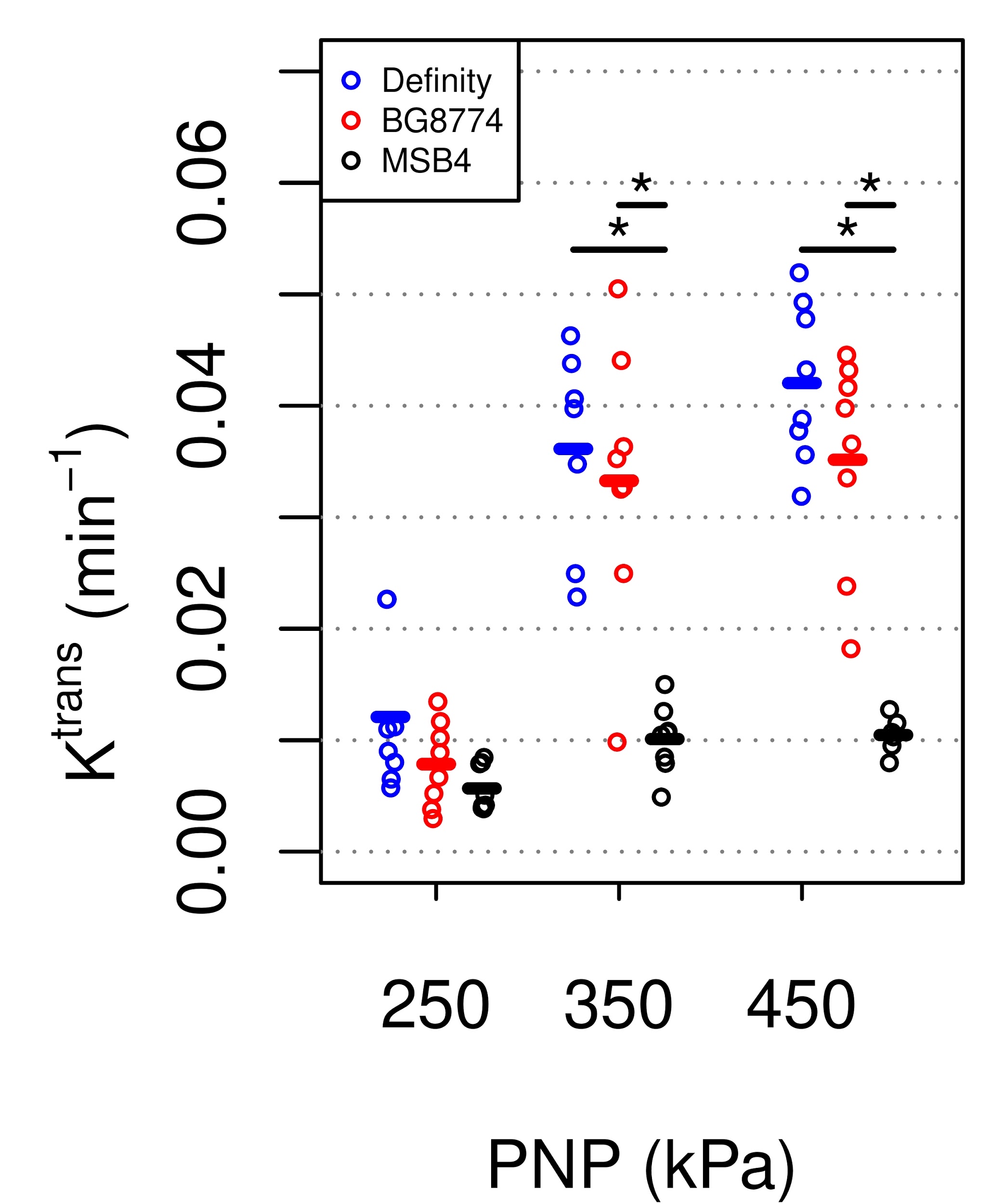


**Supplementary Figure 4: Peak negative pressure vs K^trans^.** For fixed peak negative pressures (PNPs) of 350 and 450 kPa, mean K^trans^ at targets sonicated with either Definity (p < 0.05) or BG8774 (p < 0.05) in circulation were greater than at targets sonicated with MSB4 in circulation. Mean K^trans^ values at targets sonicated with PNPs of 250 kPa, 350 kPa, and 450 kPa, respectively, were as follows: (1) Definity: 0.012 min^-1^ ± 0.007 min^-1^, 0.036 min^-1^ ± 0.009 min^-1^, 0.042 min^-1^ ± 0.007 min^-1^; (2) BG8774: 0.008 min^-1^ ± 0.004 min^-1^, 0.033 min^-1^ ± 0.012 min^-1^, 0.035 min^-1^ ± 0.010 min^-1^; (3) MSB4: 0.006 min^-1^ ± 0.002 min^-1^, 0.010 min^-1^ ± 0.003 min^-1^, 0.010 min^-1^ ± 0.002 min^-1^.


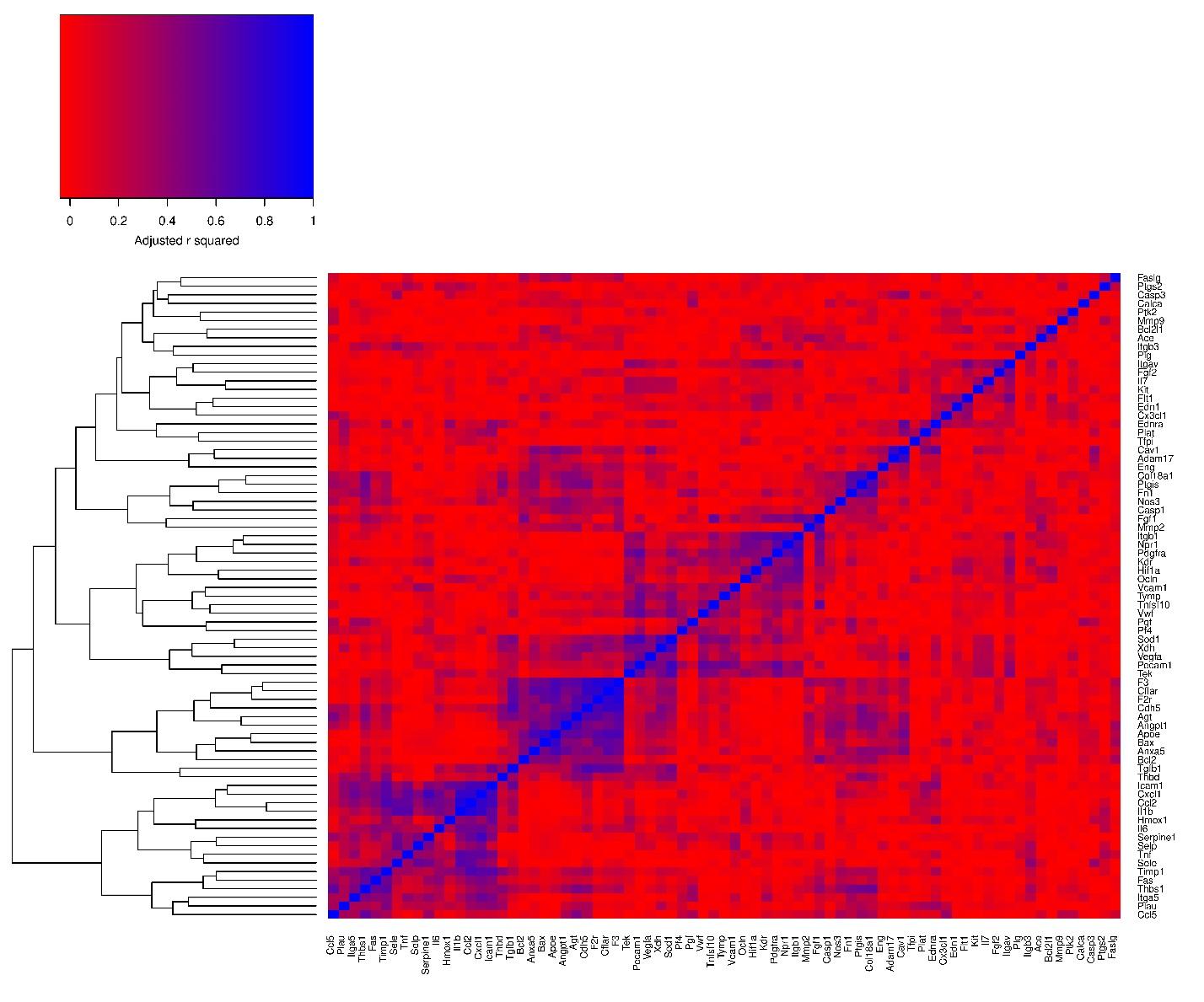


**Supplementary Figure 5: Heat map of within-sample relative expression gene-pair correlations.** Correlations in within-sample relative expression for all gene-pair combinations are displayed. Adjusted r^2^ indicates strength of correlation. Clusters of genes displaying high correlations in relative expression include: (1) *Il1b, Ccl2, Cxcl1,* and *Icam1,* (2) *F3, Cflar, F2r,* and *Cdh5,* (3) *Sod1, Xdh, Vegfa, Pecam1,* and *Tek,* (4) *Thbs1, Fas,* and *Timp1,* (5) *Col18a1, Ptgis,* and *Fn1.*


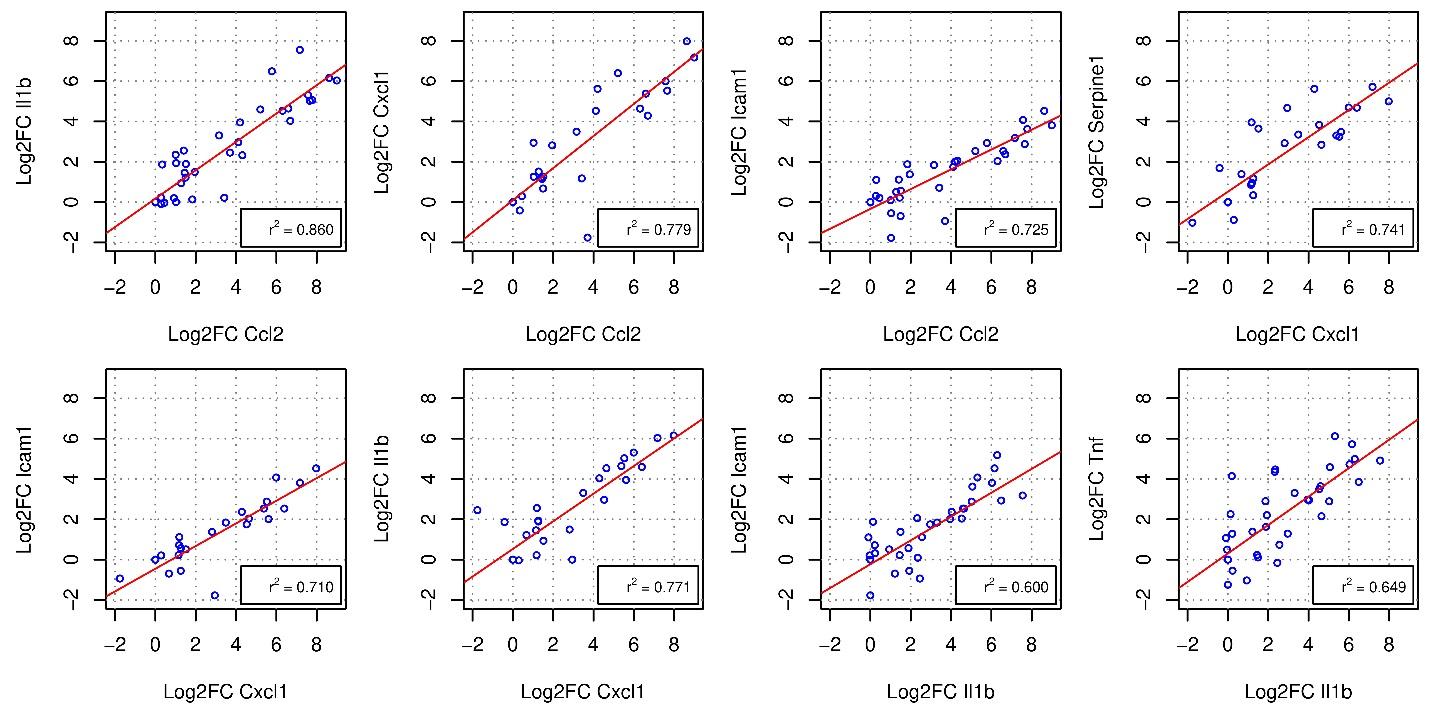


**Supplementary Figure 6: Within-sample relative expression gene-pair correlations for key inflammatory mediators.** Strong correlations in relative expression within samples were observed for several key genes involved in the regulation of inflammation, including *Il1b, Cxcl1, Ccl2, Icam1,* and *Serpine1*.
